# Clinical Spectrum, Diagnostic Work-up, and Outcomes of Neurolymphomatosis in Lymphoid Malignancies: A 10-Patient Case Series

**DOI:** 10.2147/BLCTT.S633670

**Published:** 2026-09-01

**Authors:** Safaa Alazzawi, Mohammed Abdulgayoom, Ruba Y Taha, Mohamed Abdelrazek, Wafa M Mohammed, Yahya Mulikandathil, Afaf H Al Battah, Sarah A Elkourashy, Yeslem Ekeibed, Honar Cherif

**Affiliations:** 1 Department of Hematology, National Center for Cancer Care and Research (NCCCR), Hamad Medical Corporation, Doha, Qatar; 2 Department of Radiology, National Center for Cancer Care and Research (NCCCR), Hamad Medical Corporation, Doha, Qatar; 3 Weill Cornell Medicine – Qatar (WCM-Q), Doha, Qatar; 4 College of Medicine, Qatar University, Doha, Qatar

**Keywords:** neurolymphomatosis, lymphoma, acute lymphoblastic leukemia, MRI, PET/CT, cerebrospinal fluid

## Abstract

**Purpose:**

Neurolymphomatosis (NL) is a rare infiltration of cranial nerves, nerve roots, plexuses, or peripheral nerves by malignant lymphoid cells. We aimed to describe the clinical spectrum, diagnostic work-up, treatment, and outcomes of NL in a Middle Eastern tertiary cancer center.

**Patients and methods:**

We conducted a retrospective single-center case series of patients with clinically and/or radiologically confirmed NL managed at the National Center for Cancer Care and Research, Qatar, from January 2020 to April 2026. Clinical, imaging, cerebrospinal fluid, treatment, response, and survival data were summarized descriptively.

**Results:**

Ten patients were identified; median age was 44.5 years, eight were male, eight had B-cell malignancies, and two had T-lymphoblastic leukemia/lymphoma. Three had synchronous NL at initial diagnosis, whereas seven developed NL with relapsed, refractory, or recurrent disease. MRI supported NL in 7/10 patients, cerebrospinal fluid in 4/10, and PET/CT in 3/10; none underwent direct nerve biopsy. Best neurologic response was complete in three patients, partial in four, and absent in three. Four patients were alive at censoring, and six had died of progressive disease.

**Conclusion:**

In this regional cohort, NL usually occurred with aggressive active disease and showed heterogeneous neurologic presentations. Diagnosis depended on integrating clinical findings with MRI, PET/CT, and cerebrospinal fluid studies. Although overall outcomes were poor, durable disease control was achieved in selected patients. Multicenter studies are needed to refine diagnostic pathways and treatment strategies.

## Introduction

Neurolymphomatosis (NL) refers to direct invasion of cranial nerves, spinal nerve roots, plexuses, or peripheral nerves by malignant lymphoid cells.^1–3^ It is an uncommon but increasingly recognized extranodal manifestation of lymphoma and leukemia, most often associated with aggressive B-cell lymphomas, although it has also been described in T-cell neoplasms and acute lymphoblastic leukemia.^2^,^3^

Clinical presentation is heterogeneous and largely reflects the neuroanatomic distribution of infiltration. Painful polyradiculopathy or plexopathy, mononeuropathy, mononeuritis multiplex, and cranial neuropathies are all well-described patterns.^1^,^3–5^ Because these manifestations overlap with infectious or inflammatory neuropathies, chemotherapy-related neurotoxicity, compressive syndromes, and leptomeningeal relapse, diagnosis is frequently delayed.^3^,^5^

No single test is uniformly diagnostic. MRI may demonstrate nerve enlargement, abnormal T2/STIR hyperintensity, enhancement, or denervation-related changes, whereas FDG-PET/CT can identify metabolically active neural disease and occult systemic sites that help support the diagnosis or guide biopsy.^3^,^4^,^6^,^7^ CSF cytology or flow cytometry may also be informative in selected patients, but their yield is variable and often depends on the pattern of involvement.^3^

Despite increasing recognition of NL, the evidence base remains limited. The largest international collaborative study included 50 patients and reported a median overall survival of 10 months, while subsequent institutional and aggregate studies have differed in case definition, diagnostic confirmation, and underlying malignancies.^2^,^5^,^8–15^ Published experience from the Middle East is particularly scarce.

To address this gap, we reviewed NL cases managed through routine multidisciplinary practice at a tertiary cancer center in Qatar, including both lymphoma and lymphoblastic leukemia/lymphoma and cases diagnosed without direct nerve biopsy. The primary objective was to describe the clinicopathologic spectrum and diagnostic work-up; secondary objectives were to summarize treatment, neurologic and radiologic responses, and survival, and to place these findings in the context of published series. As an exploratory descriptive case series, the study did not test a prespecified comparative hypothesis.

## Methods

This retrospective, single-center case series was conducted at the National Center for Cancer Care and Research, Hamad Medical Corporation, Qatar, in accordance with the approved study protocol and institutional confidentiality procedures for retrospective research using electronic medical records.

Eligible patients had clinically and/or radiologically confirmed cranial nerve, peripheral nerve, plexus, or nerve-root involvement by lymphoma or acute lymphoblastic leukemia, with or without concomitant CNS or leptomeningeal disease. Patients with chemotherapy-induced neuropathy, paraneoplastic neuropathy, or isolated CNS parenchymal relapse without cranial, peripheral, plexus, or root involvement were excluded.

Patients were identified through review of the institutional electronic medical record, including multidisciplinary team summaries, outpatient and inpatient hematology notes, laboratory records, and radiology reports. All patients meeting the prespecified eligibility criteria during the study period were included; no sampling was performed. Data were abstracted using a standardized collection sheet. Variables included age, sex, underlying malignancy, disease status at NL diagnosis, first neurologic presentation, neuroanatomic distribution, MRI findings, PET/CT findings, cerebrospinal fluid results, biopsy data, treatment, neurologic response, radiologic response, and survival status.

For this analysis, the date of NL diagnosis was defined as the first neurologic presentation attributable to NL or, in radiologically detected cases without a clear focal neurologic complaint, the date of first imaging documentation of neural involvement. A diagnostic modality was considered positive only when the source documentation explicitly supported NL. Best neurologic response was categorized as complete, partial, or no neurologic response. Best radiologic response was categorized as complete/near-complete response, partial response, or progressive/no response based on the latest documented MRI and/or PET/CT assessment.

Overall survival was measured from the first neurologic presentation or first imaging documentation attributable to NL to death or censoring on 15 April 2026. Because the study sought to capture all eligible cases of this rare condition, no a priori sample-size or power calculation was performed. Analyses were descriptive and are presented as medians (range) or numbers (percentage).

## Results

### Patient Characteristics, Disease Status at NL Diagnosis, and Pattern of Neurolymphomatosis

**Figure 1:**
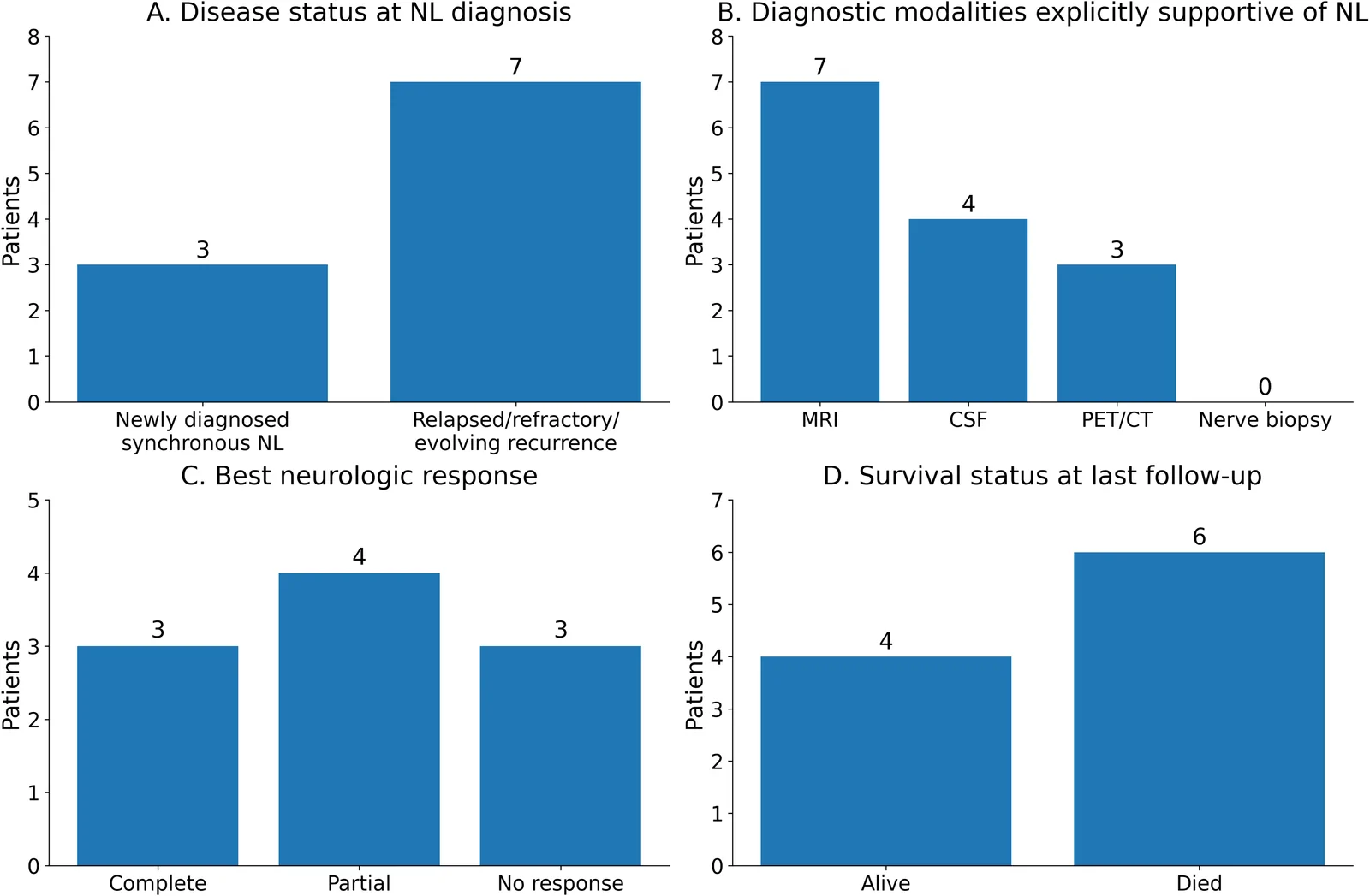
Diagnostic profile and outcomes of the 10 included patients. (**A**) Disease status at neurolymphomatosis (NL) diagnosis: newly diagnosed with synchronous NL (n=3) and relapsed, refractory, or evolving recurrent disease (n=7). (**B**) Diagnostic modalities explicitly supportive of NL: MRI (n=7), cerebrospinal fluid (CSF; n=4), PET/CT (n=3), and direct nerve biopsy (n=0); modalities were not mutually exclusive. (**C**) Best neurologic response: complete (n=3), partial (n=4), and no response (n=3). (**D**) Survival status at last follow-up: alive (n=4) and deceased (n=6).

Ten patients met the study definition for neurolymphomatosis during the study period (Table 1 and Table 2; Figure 1). The cohort was predominantly male (8/10), with a median age of 44.5 years (29–60 years). Underlying malignancies were heterogeneous but mainly aggressive lymphoid neoplasms: eight patients had B-cell malignancies, including Burkitt lymphoma, diffuse large B-cell lymphoma, and high-grade B-cell lymphoma, whereas two had T-lymphoblastic leukemia/lymphoma.

**Table 1:** Baseline Clinic-Pathologic Characteristics, Disease Status at Neurolymphomatosis Diagnosis, First Neurologic Presentation, Neuroanatomic Distribution, and Diagnostic Features

Case	Age, Sex	Underlying Malignancy	Disease Status at NL Diagnosis	First Neurologic Presentation or First Imaging Documentation (Used as NL Date)	Neuroanatomic Distribution of NL	Diagnostic Modalities Explicitly Supportive of NL
1	29, M	Burkitt lymphoma	Newly diagnosed, treatment-naive advanced disease with synchronous secondary CNS involvement	May 2025: progressive low back pain radiating to buttocks/thighs followed by bilateral lower-limb weakness/paraplegia and neurogenic bladder	Cauda equina / lumbosacral root involvement with concomitant secondary CNS disease	MRI positive: multilevel vertebral/epidural soft tissue with cauda equina compression at S1-S2
2	60, M	Large B-cell lymphoma with MYC and BCL6 translocation	First relapsed disease after prior complete remission; later multiply relapsed/refractory	February 2023: left hypoglossal nerve palsy	Cranial nerve NL (hypoglossal; later sixth cranial nerve palsy at relapse)	CSF positive: monotypic CD19/CD20-positive B cells consistent with relapsed disease
3	36, M	T-lymphoblastic leukemia/lymphoma	Evolving relapse after frontline therapy, with overt marrow/CSF relapse confirmed shortly afterward	July 2025: left shoulder/back pain followed by facial weakness, dysarthria, dysphagia, headache, left arm pain, and hand numbness	Multifocal NL involving cranial nerves, cervical roots, and brachial plexus	MRI positive; CSF positive; PET/CT positive
4	54, F	High-grade B-cell lymphoma, CD10-positive	Primary refractory / suboptimal response disease at time of NL development	July 2024: back pain radiating to both legs with weakness and numbness	Lumbosacral foraminal / root involvement	MRI positive: retroperitoneal/perivertebral soft tissue from L3 to S1 with right L5-S1 neural foraminal obliteration and small intraspinal extension
5	53, M	Large B-cell lymphoma with secondary CNS/leptomeningeal involvement	Newly diagnosed disease with synchronous secondary CNS/leptomeningeal localization	March 2024: diplopia with left sixth nerve palsy, followed by nasal regurgitation, dysphagia, muffled voice, left shoulder pain, and left arm weakness/numbness	Cranial neuropathy-predominant disease with associated left upper-limb neurologic symptoms in the setting of leptomeningeal/paraspinal involvement	CSF positive: approximately 31% CD10-positive kappa monotypic B cells consistent with CNS involvement by mature B-cell neoplasm
6	30, M	T-lymphoblastic leukemia/lymphoma	CNS relapse during treatment course	July 2025: right-eye vision loss with left-eye blurring	Bilateral peri-optic / cranial nerve involvement	MRI positive; CSF positive: bilateral optic perineuritis/peri-optic leukemic infiltration on MRI and CSF flow cytometry consistent with CNS relapse
7	40, M	High-grade B-cell lymphoma, NOS	Relapsed/refractory active disease on third-line therapy	July 2025: progressive right-arm swelling and weakness with enlarging axillary mass	Right brachial plexus / peripheral nerve involvement	MRI positive: right axillary mass abutting brachial plexus and especially radial nerve
8	49, M	CD20-negative diffuse large B-cell lymphoma, NOS, activated B-cell type, triple expressor	Newly diagnosed disease with synchronous radiologic T10 neurolymphomatosis	December 2025: no focal neurologic symptoms; T10 nerve involvement recognized on staging PET/CT	T10 nerve / thoracic root involvement in the setting of stage IV-E extranodal disease	PET/CT positive: T10 neurolymphomatosis documented on staging PET/CT
9	33, F	High-grade B-cell lymphoma / diffuse large B-cell lymphoma, NOS	Primary refractory disease after frontline therapy	November 2024: new focal uptake in the right S1 sacral foramen on end-of-treatment PET/CT, with corresponding sacral neural foraminal lesion on MRI	Right sacral nerve-root / lumbosacral foraminal involvement	MRI positive; PET/CT positive: right sacral wing lesion extending into the S1-S2 neural foramen with focal PET uptake likely representing NL
10	59, M	Transformed high-grade B-cell lymphoma / diffuse large B-cell lymphoma, GCB phenotype	Recurrent paraspinal lymphoma at relapse; later transformed chemo-refractory disease	October 2024: upper back pain with thoracic paraspinal mass encroaching the left T5-T6 neuroforamina	Thoracic paraspinal / neuroforaminal root involvement	MRI positive: paraspinal soft-tissue disease extending from T4 to T7 with encroachment on the left T5-T6 neuroforamina

**Abbreviations**: CNS, central nervous system; CSF, cerebrospinal fluid; MRI, magnetic resonance imaging; NL, neurolymphomatosis; PET/CT, positron emission tomography/computed tomography.

**Table 2:** Treatment Approaches, Neurologic and Radiologic Responses, and Survival Outcomes

Case	NL-Directed/Lymphoma-Directed Treatment	Best Neurologic Response	Best Radiologic Response	Survival Outcome from First Neurologic Presentation	Status at Last Follow-Up
1	R-CODOX-M/IVAC with intrathecal therapy; rehabilitation	Partial	Complete / near-complete metabolic response	Alive beyond approximately 10 months	Alive
2	Prior HD-MTX, R-ICE, and CAR-T; later best supportive care with planned intrathecal MTX during terminal relapse	No response	Progressive disease / no meaningful NL-specific radiologic response	Approximately 11 months	Deceased of disease progression
3	High-dose methotrexate, intrathecal therapy twice weekly until CSF clearance, nelarabine-based salvage	Partial	Complete radiologic/metabolic resolution of previously FDG-avid NL	Approximately 2 months	Deceased of disease progression
4	Prior CHOP-based therapy; salvage R-DHAP planned	No response	No response / progressive refractory disease	Less than 1 month	Deceased of disease progression
5	R-CODOX-M/IVAC, MATRIX consolidation, WBRT and sacral radiotherapy, ibrutinib, glofitamab, later high-dose methotrexate; subsequently best supportive care	Partial	Complete / near-complete response followed by later CNS progression	Approximately 19 months	Deceased of disease progression
6	High-dose methotrexate, repeated intrathecal MTX, CNS radiotherapy, followed by matched-sibling allogeneic HSCT	Complete	Complete radiologic response / remission	Alive beyond approximately 8 months	Alive on 24 March 2026
7	GemOx, epcoritamab, palliative axillary radiotherapy, corticosteroids, supportive care	Partial	Partial local response with reduction of axillary mass after radiotherapy	Approximately 1 month	Deceased of disease progression
8	R-CODOX-M/IVAC x4 courses	Complete	Complete metabolic response	Alive beyond approximately 4 months	Alive on 6 April 2026
9	Bridging polatuzumab + rituximab and sacral radiotherapy followed by liso-cel CAR-T; surveillance/supportive care	Complete	Complete metabolic response / maintained remission with stable post-treatment changes	Alive beyond approximately 16 months	Alive with maintained CMR on 15 April 2026
10	Bendamustine-obinutuzumab, ICE, autologous HSCT; later Pola-GemOx and best supportive care during terminal relapse	No response	Progressive disease	Approximately 18 months	Deceased of disease progression

**Notes**: NL date was defined as the first neurologic presentation attributable to cranial nerve, peripheral nerve, plexus, or root infiltration, or the date of first imaging documentation when neural involvement was detected radiologically without a clear focal neurologic complaint. Diagnostic yield was counted only when MRI, PET/CT, CSF, or biopsy explicitly supported NL. Lymph-node or extranodal biopsy confirming lymphoma was not counted as direct NL confirmation unless nerve or root tissue itself was sampled. Overall survival was measured from first neurologic presentation or first imaging documentation to death or censoring on 15 April 2026.

At the time NL was recognized, three patients had newly diagnosed disease with synchronous NL, whereas seven had relapsed, refractory, or evolving recurrent disease. The latter group included first relapse after a previous remission, primary refractory disease after frontline therapy, multiply relapsed high-grade B-cell lymphoma, recurrent paraspinal lymphoma that later transformed, and two cases of T-lymphoblastic leukemia/lymphoma with CNS relapse. Thus, NL was usually associated with biologically aggressive or treatment-resistant disease, although it also occurred at initial diagnosis.

Neurologic involvement was diverse and often multifocal. Cranial nerve manifestations included hypoglossal and sixth-nerve palsies, facial weakness with dysarthria and dysphagia, vocal cord paralysis, and bilateral visual loss or blurring related to peri-optic disease. Other presentations included cauda equina syndrome, lumbosacral radiculopathy, cervical root and thoracic neuroforaminal involvement, and brachial plexus or radial nerve infiltration. One patient had asymptomatic T10 nerve involvement detected on staging PET/CT, while another had simultaneous cranial nerve, cervical root, and brachial plexus disease.

### Diagnostic Work-Up

**Figure 2:**
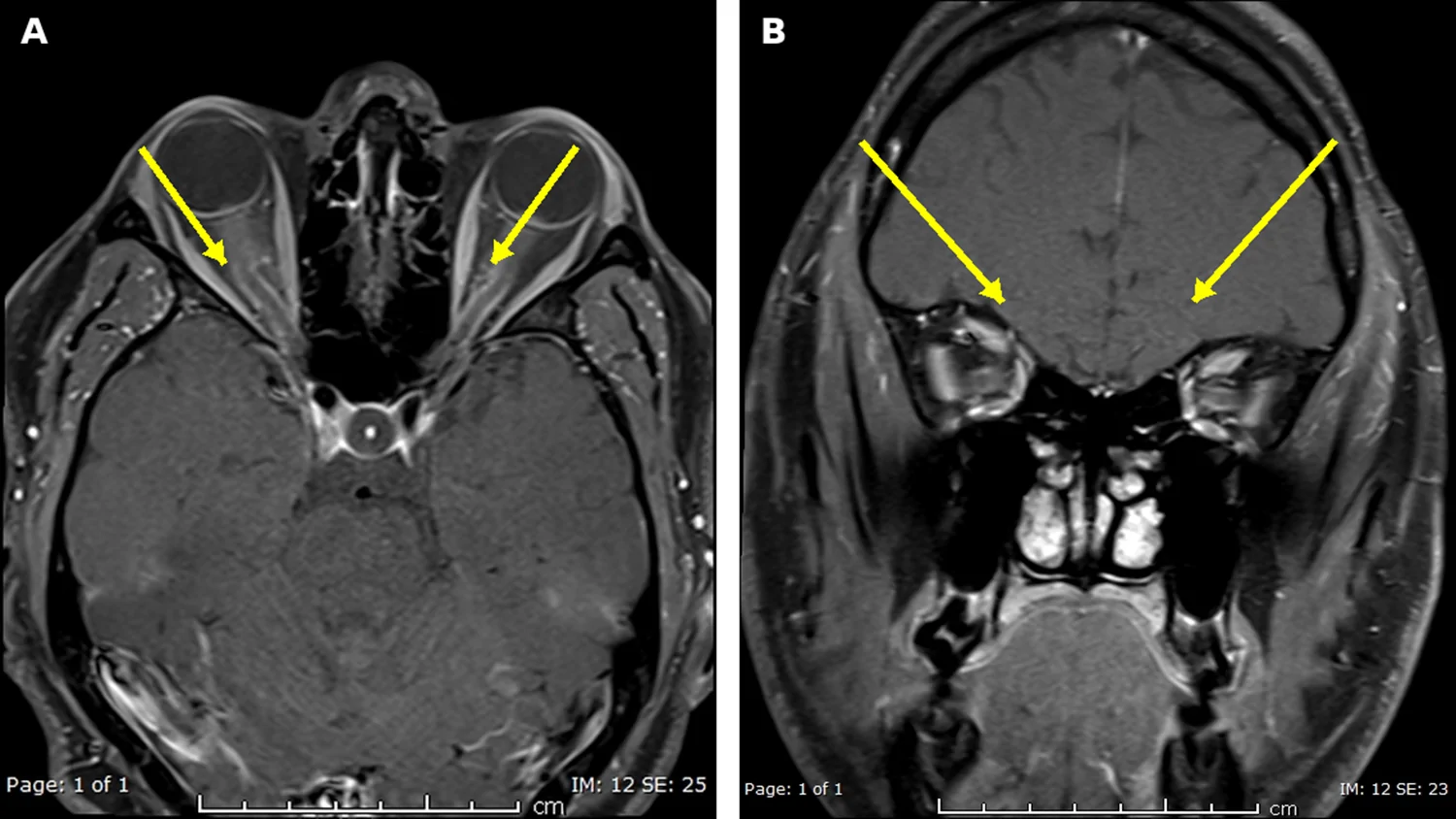
MRI features of bilateral peri-optic neurolymphomatosis in a patient with T-lymphoblastic leukemia/lymphoma. (**A**) Axial and (**B**) coronal post-contrast fat-saturated T1-weighted brain MRI images show uniform thickening and enhancement of the peri-optic nerve meningeal sheaths (yellow arrows).

**Abbreviations**: CSF, cerebrospinal fluid; MRI, magnetic resonance imaging; NL, neurolymphomatosis; PET/CT, positron emission tomography/computed tomography.

MRI explicitly supported NL in seven of ten patients (Table 1). Findings included cauda equina compression, neural foraminal obliteration with root extension, cranial nerve thickening and enhancement, cervical root thickening, bilateral optic perineuritis suspicious for leukemic infiltration (Figure 2), an axillary mass abutting the brachial plexus and radial nerve, a sacral wing lesion extending into the S1-S2 neural foramen, and thoracic paraspinal disease encroaching on the T5-T6 neuroforamina. PET/CT supported NL in three patients: one with FDG-avid involvement of the left C4 root and right brachial plexus, one with T10 nerve involvement identified at staging, and one with focal uptake in the right S1 sacral foramen corresponding to a neural foraminal lesion on MRI.

Cerebrospinal fluid studies supported NL in four patients: two with large B-cell lymphoma had monotypic CD19/CD20-positive B cells, and two with T-lymphoblastic leukemia/lymphoma had flow-cytometric evidence of leukemic CNS involvement. No patient underwent direct nerve biopsy; diagnosis therefore relied on the combined clinical, imaging, cerebrospinal fluid, and disease context.

### Treatment and Outcomes

Treatment was individualized according to the underlying malignancy and disease setting (Table 2). All patients received systemic disease-directed therapy, often combined with high-dose or intrathecal methotrexate, radiotherapy, salvage chemotherapy, cellular therapy, or transplantation. One patient with secondary CNS/leptomeningeal large B-cell lymphoma received R-CODOX-M/IVAC followed by MATRIX consolidation, whole-brain and sacral radiotherapy, ibrutinib, glofitamab, and later palliative high-dose methotrexate after progression. A patient with newly diagnosed CD20-negative diffuse large B-cell lymphoma and T10 nerve involvement achieved a complete metabolic response after four courses of R-CODOX-M/IVAC. Another patient with primary refractory sacral nerve-root lymphoma received bridging polatuzumab plus rituximab and sacral radiotherapy before liso-cel CAR-T therapy and remained in metabolic remission at last follow-up. By contrast, a patient with transformed high-grade B-cell lymphoma and thoracic neuroforaminal disease relapsed rapidly after autologous transplantation and died despite Pola-GemOx and supportive care.

Best neurologic response was complete in three patients, partial in four, and absent in three. Best radiologic response was complete or near-complete in six patients, partial in one, and progressive or absent in three. At last follow-up, the four surviving patients included one with Burkitt lymphoma and cauda equina involvement in remission after intensive chemotherapy and intrathecal treatment; one with sacral nerve-root lymphoma in complete metabolic remission after CAR-T therapy; one with T-lymphoblastic leukemia/lymphoma and bilateral peri-optic involvement in remission after high-dose methotrexate, intrathecal therapy, radiotherapy, and allogeneic transplantation; and one with CD20-negative diffuse large B-cell lymphoma and T10 nerve involvement who achieved an end-of-therapy complete metabolic response after R-CODOX-M/IVAC. The other six patients had refractory or progressive disease and died despite salvage treatment.

At censoring on 15 April 2026, four of ten patients were alive and six had died of progressive disease. Among the six deceased patients, observed survival from first neurologic presentation or first imaging documentation attributable to NL ranged from less than 1 month to approximately 19 months. Because four patients remained alive after approximately 4 to 16 months of follow-up, a robust median overall survival for the entire cohort was not estimated. The shortest survival occurred in patients with rapidly progressive refractory disease and poor functional status at presentation, whereas longer survival was observed in patients who initially responded before later CNS or systemic progression. Overall, survival was heterogeneous but generally poor when NL developed in the setting of aggressive disease biology.

## Discussion

This series expands the limited Middle Eastern evidence on NL and reflects routine multidisciplinary practice in a cohort that included both B-cell lymphomas and T-lymphoblastic leukemia/lymphoma without requiring nerve-biopsy confirmation. Most patients (70%) developed NL with relapsed, refractory, or recurrent disease, while 30% presented synchronously at initial diagnosis. The proportion with synchronous disease was close to the 26% reported in the 50-patient international collaborative study but lower than the 52% classified as primary NL in a biopsy-proven Mayo Clinic cohort, likely reflecting differences in case definition and referral selection.^2^,^10^

Clinical presentations spanned cranial neuropathies, peri-optic disease, radiculopathy, cauda equina syndrome, brachial plexus and peripheral nerve infiltration, thoracic neuroforaminal disease, and asymptomatic neural involvement detected at staging or restaging. This heterogeneity is consistent with classic and contemporary descriptions of NL.^1^,^3^,^10^,^14^,^15^ Diagnostic delay remains a major concern: a recent 22-patient series reported a median interval of 3 months from symptom onset to diagnosis and found that earlier use of MRI or FDG-PET was associated with shorter diagnostic intervals.^12^ New focal or multifocal deficits, severe neuropathic pain, or unexplained nerve-root or plexus abnormalities in patients with lymphoma or lymphoblastic leukemia should therefore prompt consideration of NL rather than automatic attribution to treatment toxicity.

MRI was the principal supportive test in our cohort, identifying NL in 70% of patients. This was comparable to the 77% positivity reported in the international collaborative series and to abnormal MRI findings in 8 of 9 evaluable patients in a 2021 single-center cohort.^2^,^5^ Cerebrospinal fluid was supportive in 40%, the same proportion reported in the international series.^2^ PET/CT supported NL in 30%, lower than the yields described in pathology-selected or dedicated imaging studies.^2^,^6^,^10^,^11^ This lower proportion may reflect our conservative, report-based definition and the frequent use of PET/CT for systemic staging rather than targeted evaluation of neurologic symptoms. No patient underwent direct nerve biopsy, highlighting that tissue confirmation may be invasive or anatomically unsafe and that diagnosis often depends on integration of the clinical phenotype, imaging, cerebrospinal fluid findings, and underlying disease context.^2^,^3^

Treatment was heterogeneous because no standard NL-specific regimen exists and therapy must be adapted to the underlying malignancy and disease setting. Neurologic improvement occurred in seven patients. Although this exceeds the 46% response reported in the international series and the three improvements among ten patients in another single-center cohort, direct comparison is limited by differences in case selection, response definitions, and treatment.^2^,^5^ Six patients died of progressive disease, consistent with the poor survival reported in previous studies, including a historical median overall survival of 10 months and a median of 18 months in a recent individual-patient systematic analysis.^2^,^11^ Durable disease control after CAR-T therapy or after high-dose methotrexate, radiotherapy, and allogeneic transplantation was observed in selected patients, but these findings are hypothesis-generating and cannot establish the effectiveness of any individual treatment.

The study has several limitations. Its retrospective, single-center design and small sample increased the risk of referral and ascertainment bias and precluded formal comparative analysis. Reliance on electronic documentation may have missed incompletely recorded cases, while the requirement for explicit support in source reports may have underestimated the diagnostic contribution of PET/CT or cerebrospinal fluid studies. Neurologic and radiologic responses were derived from routine documentation rather than validated NL-specific criteria. In radiologically detected cases with minimal or no focal symptoms, the date of first imaging documentation was used as the NL date. Finally, heterogeneous treatment and limited follow-up prevented causal inference and reliable estimation of median overall survival.

Taken together, these findings reinforce the value of early multidisciplinary assessment. Collaboration among hematology, neurology or neuro-oncology, neuroradiology, nuclear medicine, and pathology can help distinguish NL from treatment toxicity, infection, inflammatory neuropathy, compression, or leptomeningeal relapse and guide the selective use of MRI, PET/CT, cerebrospinal fluid assessment, and biopsy. Although our data cannot show that earlier diagnosis improves survival, prompt evaluation may reduce avoidable diagnostic delay and allow timely disease-directed management.^12^ The peri-optic involvement observed in T-lymphoblastic leukemia/lymphoma, asymptomatic neural disease detected on staging PET/CT, and durable control after cellular therapy or transplantation are notable observations that merit further study in larger multicenter cohorts.

## Conclusion

In this 10-patient series, NL most often occurred with relapsed or refractory lymphoid malignancy and involved multiple neuroanatomic sites. Diagnosis was usually established by integrating clinical findings with MRI, PET/CT, and cerebrospinal fluid results rather than by direct nerve biopsy. Outcomes were generally poor, although durable disease control was achieved in selected patients. Prompt multidisciplinary assessment is warranted when patients with lymphoid malignancies develop unexplained focal or multifocal neurologic abnormalities. Larger multicenter studies are needed to establish optimal diagnostic and therapeutic strategies.

## Data Availability

The data supporting the findings of this study are available from the corresponding author upon reasonable request. Due to institutional policies and ethical restrictions regarding patient confidentiality, raw data are not publicly available. Access to de-identified data may be granted to qualified researchers subject to approval by the Medical Research Center at Hamad Medical Corporation.
